# Electrically controllable active plasmonic directional coupler of terahertz signal based on a periodical dual grating gate graphene structure

**DOI:** 10.1038/s41598-021-90876-2

**Published:** 2021-06-01

**Authors:** Mikhail Yu. Morozov, Vyacheslav V. Popov, Denis V. Fateev

**Affiliations:** 1 Kotel’nikov Institute of Radio Engineering and Electronics (Saratov Branch), RAS, Saratov, Russia 410019; 2 National Research Saratov State University, Saratov, Russia 410012

**Keywords:** Nanophotonics and plasmonics, Terahertz optics

## Abstract

We propose a concept of an electrically controllable plasmonic directional coupler of terahertz signal based on a periodical structure with an active (with inversion of the population of free charge carriers) graphene with a dual grating gate and numerically calculate its characteristics. Proposed concept of plasmon excitation by using the grating gate offers highly effective coupling of incident electromagnetic wave to plasmons as compared with the excitation of plasmons by a single diffraction element. The coefficient which characterizes the efficiency of transformation of the electromagnetic wave into the propagating plasmon has been calculated. This transformation coefficient substantially exceeds the unity (exceeding 6 in value) due to amplification of plasmons in the studied structure by using pumped active graphene. We have shown that applying different dc voltages to different subgratings of the dual grating gate allows for exciting the surface plasmon in graphene, which can propagate along or opposite the direction of the structure periodicity, or can be a standing plasma wave for the same frequency of the incident terahertz wave. The coefficient of unidirectionality, which is the ratio of the plasmon power flux propagating along (opposite) the direction of the structure periodicity to the sum of the absolute values of plasmon power fluxes propagating in both directions, could reach up to 80 percent. Two different methods of the plasmon propagation direction switching are studied and possible application of the found effects are suggested.

## Introduction

Controlling the propagation the plasma waves (plasmons) as well as switching of plasmon propagation direction is important for designing integrated plasmonic nanocircuits. Noble metals are suitable for exciting and controlling plasmons in the visible and near-infrared frequency range. But at terahertz (THz) frequencies, plasmons on the surfaces of noble metals are weakly confined. Terahertz plasmonic devices can be implemented on other materials, graphene for example. Graphene is a unique material with a zero energy gap and linear spectrum of electrons at THz frequency range^1^. As was shown in^2,3^, the momentum relaxation time of charge carriers in graphene reaches the value of 2 ps at room temperature. Hence, graphene is a promising platform for excitation and manipulation of plasmons in the THz frequency range^4^. Possibility of population inversion of free charge carriers in graphene at THz frequencies^5^ along with the possibility of excitation of highly confined graphene plasmons^6^ resulted in proposals of THz graphene lasers^7,8^ and plasmonic amplifiers^9,10^ which may be used for signal processing in THz integrated nanocircuits in the subwavelength regime^11^.

There are several methods for excitation of plasmons in graphene. It can be the excitation of surface plasmon polaritons propagating along the graphene using diffraction of incident THz wave on a grating gate^12^, a single metal gate^13^ or a thin slit in a metal gate^14^ which are placed above graphene. One may use the diffraction of external waves on the cantilever of a scanning near-field microscope^15^. However, the diffraction of THz wave on a single diffraction element couples THz wave to plasmons in graphene with rather low efficiency. Another conventional method for exciting plasmon polaritons in graphene is using the prism coupling^16,17^ in the Otto or Kretschmann configurations^18,19^. In order to create plasmonic splitters or directional couplers one has to make the plasmon propagate in a desired direction. Diffraction of incident wave on a periodical grating gate with a symmetric unit cell demonstrates high efficiency of transformation of incident wave into standing plasma wave^20,21^ but does not allow for launching the unidirectionally travelling plasmon. Creation of travelling plasmon by using attenuated total reflection methods suffers from strong effect of the angle of incidence on the transformation of incident wave into travelling plasmon. To enhance the transformation efficiency of an incident electromagnetic wave into a propagating plasmon, a periodical graphene structure with a dual-grating gate having an asymmetric unit cell was proposed and theoretically investigated in^22,23^. Spatially asymmetric plasmon structures can detect THz radiation^24–26^. Excitation and amplification of THz plasmons using diffraction of incident wave on a periodical grating gate were experimentally observed in^27,28^.

Tunable switches, splitters and couplers are of great importance in optical communication systems and integrated nanocircuits. Graphene-based switch in the near infrared frequency range and its fabrication and operation principles were demonstrated in^29^, where the method of switching the graphene conductance between high and low conductance states using appropriate bias voltage pulses was proposed. Trapping the plasmon polaritons on the graphene surface within a broadband frequency range was demonstrated theoretically and numerically in^30^. A tunable Y-branch graphene plasmonic switch operating at the wavelength of 1.55 mm was proposed and analyzed analytically and numerically in^31^. Proposed in that paper plasmonic switch supports transverse magnetic and transverse electric graphene plasmons whose propagation characteristics could be controlled by modulating the external electric field and the temperature of graphene. Plasmonic splitter based on two different metal–insulator-metal waveguides with periodic grooves and a subwavelength slit was proposed and theoretically studied in^32^. Terahertz tunable plasmonic directional coupler based on a graphene and a thin metal film with a nanoscale slit was proposed in^14^. The possibility of switching the plasmon propagation direction applying different voltages to the graphene layer was numerically shown in that paper.

**Figure 1:**
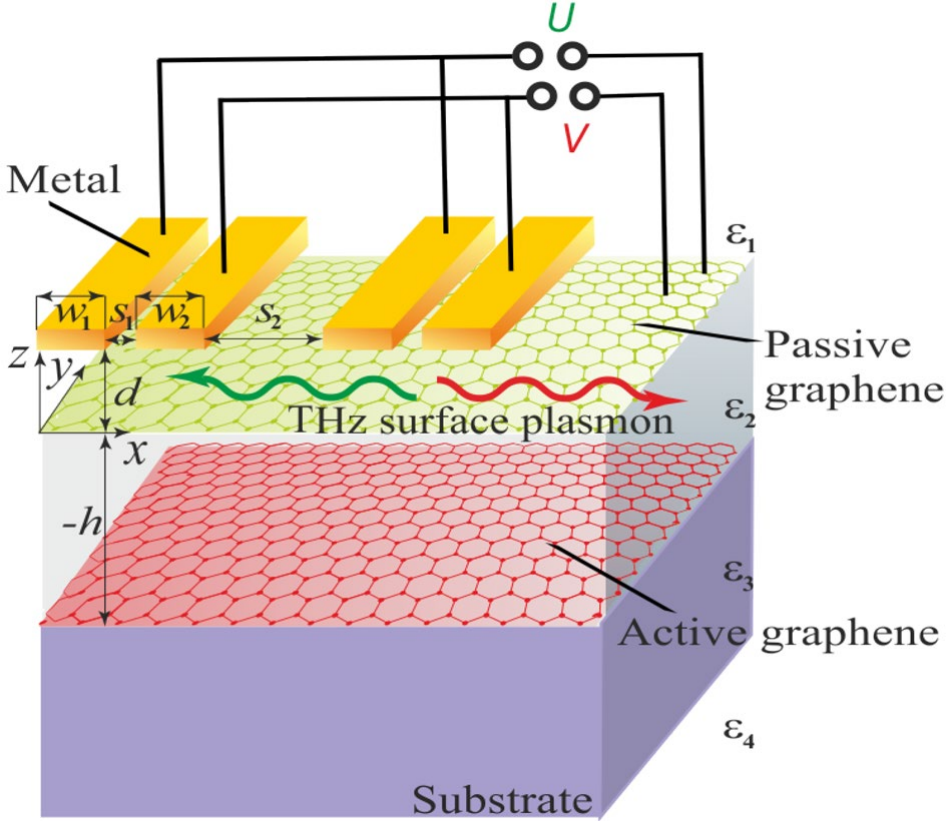
Schematic view of the structure under consideration (two unit cells of the periodic structure are shown).

In this paper, we theoretically investigate electrically controllable THz plasmonic directional coupler based on a periodic graphene structure with a dual grating gate, termed dual grating gate graphene heterostructure (DGGGH), which is schematically shown in Fig. 1. The dual grating gate consists of two interdigitated comblike subgratings, the finger of each are connected electrically. Active graphene with population inversion of free charge carriers is deposited on dielectric or semiconductor substrate and covered by an insulating layer. To pump this graphene layer, one can use optical pumping^33^ or injection pumping^34^, or diffusion pumping proposed in^35,36^. Passive graphene (without population inversion) is overlayed upon the insulating layer separating the graphene layers. This passive graphene is screened by periodic metal dual grating gate that is placed over the structure. Incident THz electromagnetic wave with the electric field polarized across the grating gate fingers diffracts on the periodic gate and excites transverse magnetic (TM) plasmons (the magnetic field of the plasmon is polarized in the *y*-direction) in both graphene layers. We have studied the structure with equal widths of the grating-gate fingers in both subgratings comprising the dual grating gate. Thus, the structure under consideration has a geometrically symmetric unit cell. It is known that for effective excitation of the propagating plasmon one needs an asymmetry in the unit cell of the structure^21^. Applying constant dc voltage *U* or *V* between the gate and upper graphene layer, one can change the Fermi energy of the free charge carriers in the gated regions of the upper graphene by the value of *eU* or *eV*, respectively, where *e* is the electron charge. Change of the equilibrium electron concentration in graphene applying the constant voltage to the gate subgratings of the dual grating gate structures is experimentally and theoretically described in^27,28^. As was shown in^37^, the voltage applied to the gate almost does not affect the concentration of the free charge carriers in the lower graphene layer. Carrier concentration in the lower graphene could be changed if only the upper graphene concentration is depleted that was not realized for applied voltage values used in our calculations. Applying different dc voltages to different subgratings of the dual grating gate one can create spatial asymmetry in the unit cell of the studied structure (forming different Fermi energy levels under different gate subgratings) which allows for switching of plasmon propagation direction. Active graphene allows for amplification of the propagation plasmon.

## Results

**Figure 2:**
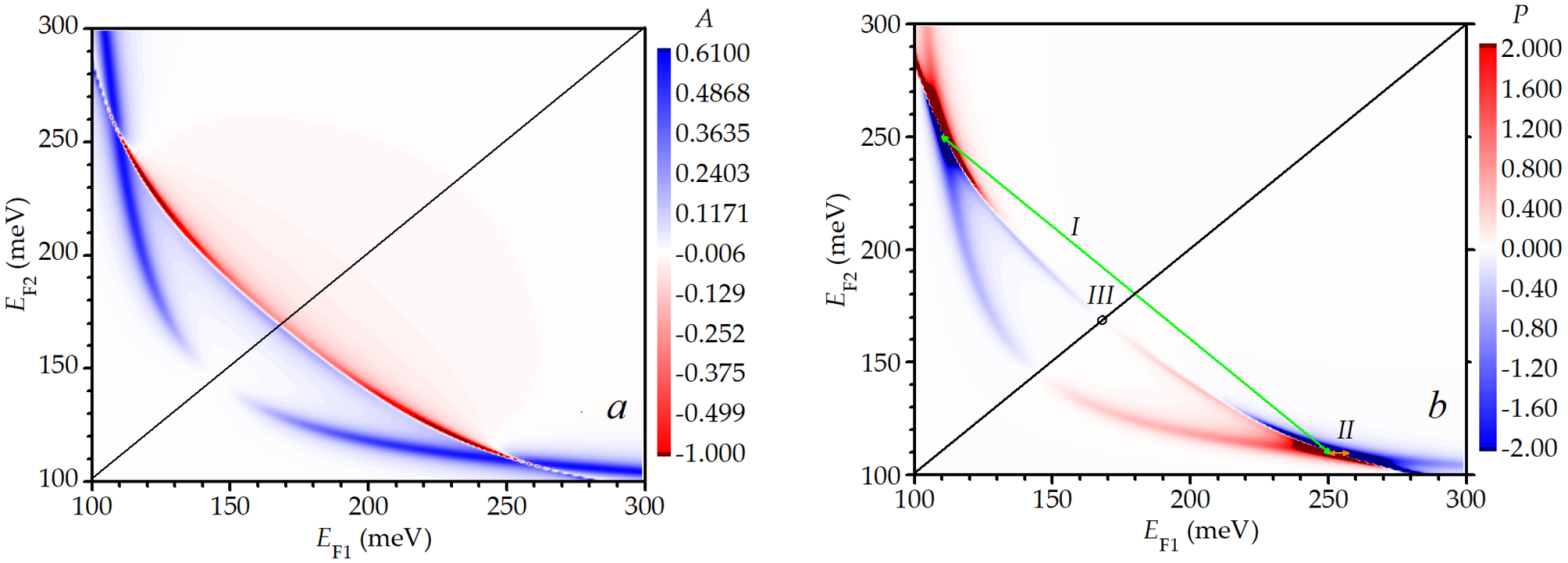
Absorption coefficient of the incident electromagnetic wave (**a**) and transformation coefficient of the incident wave into the propagating plasmon. (**b**) in the DGGGH in dependence on the Fermi energy values in the gated regions of the upper graphene. Plasmon direction switches of different types are denoted by *I* and *II*. Excitation of the standing plasma wave for symmetric DGGGH is shown by point *III.*

**Figure 3:**
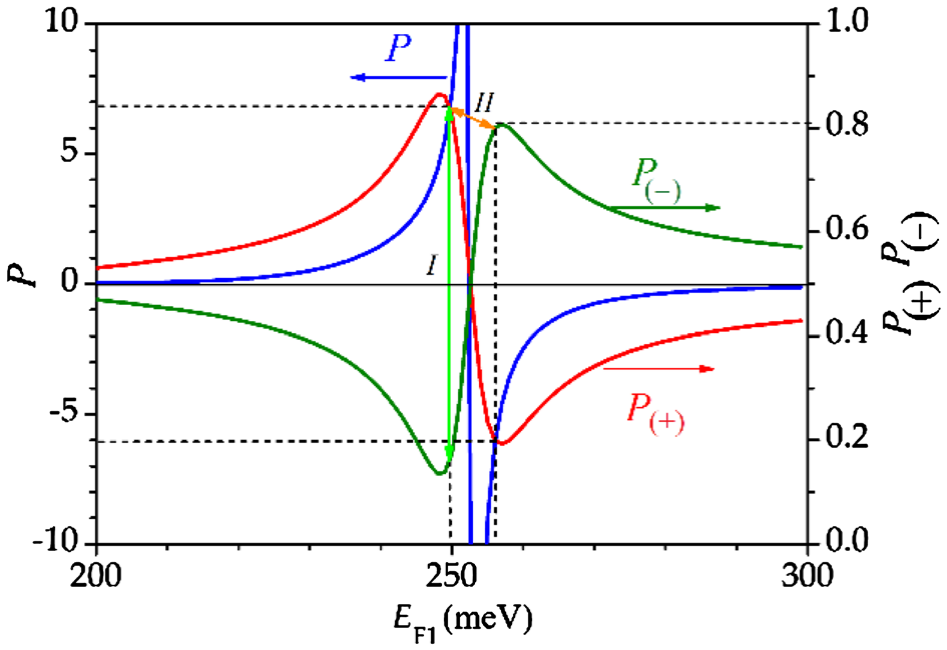
Transformation coefficient of the incident electromagnetic wave into the propagating plasmon *P* and positive $P_{( + )}^{{}}$ and negative $P_{( - )}^{{}}$ unidirectional coefficients in the DGGGH in dependence on the Fermi energy value $E_{{{\text{F1}}}}$ under one of subgratings for a fixed Fermi energy $E_{{{\text{F2}}}} = 110$ meV under the gates of the other subgrating. Plasmon direction switches of different types are denoted by *I* and *II*.

The coefficients of absorption of the electromagnetic wave and transformation of this incident wave into the propagating plasmon in the studied DGGGH are presented in Fig. 2(a and b panels). Numerical calculations were performed for the structure with the following parameters, allowing for THz plasmon excitation (notations are presented in Fig. 1): both gate subgrating fingers widths are $w_{1} = w_{2} = 0.3$µm, gaps between the subgrating fingers are $s_{1} = 0.1$ µm and $s_{2} = 0.5$ µm, the distance between the gate and the upper graphene is equal to $d = 30$ nm, the distance between the graphene layers (the insulating layer thickness) is $h = 30$ nm, temperature of free charge carriers in the graphene layers is *T* = 300 K (assumed the same in both graphene layers), and free carrier momentum relaxation time is $\tau = 1$ ps ^38^. The quasi-Fermi energy value in the lower (active) graphene (formula for the conductivity of pumped graphene is given in the supplemental material) was taken to be $\varepsilon_{F} = 100$ meV, which corresponds to the negative real part of the active graphene conductivity^5,7^. The frequency was fixed at $f = 5$ THz for calculations results of which are presented in Figs. 2 and 3. This frequency belongs to the frequency region with negative real part the active graphene conductivity^5, 7^.

The coefficient of absorption of the incident wave in the DGGGH is defined as $A = 1 - {{\left( {S^{{\text{R}}} + S^{{\text{T}}} } \right)} \mathord{\left/ {\vphantom {{\left( {S^{{\text{R}}} + S^{{\text{T}}} } \right)} {S^{{{\text{inc}}}} }}} \right. \kern-\nulldelimiterspace} {S^{{{\text{inc}}}} }}$ where $S^{{\text{R}}} ,$ $S^{{\text{T}}} ,$ and $S^{{{\text{inc}}}}$ are the power fluxes of the reflected, transmitted and incident THz electromagnetic wave, respectively. The Fermi energy value in the upper graphene under the gate fingers subjected to the voltage *U* (see Fig. 1) we denote as $E_{{{\text{F1}}}} ,$ while $E_{{{\text{F2}}}}$ is the Fermi energy value in the upper graphene under the gate fingers with the voltage *V*. The map is symmetric regarding to the absorption coefficient with respect to diagonal from left-bottom to right-top regions of the map due to the symmetry of the structure for interchanging the voltage values in the subgratings. One can see two plasmon modes on the map which intercross for certain Fermi energy values. Negative absorption coefficient values in some regions of the map (that means the plasma wave amplification) are caused by the presence of the lower active graphene.

The coefficient of transformation of the electromagnetic wave into a propagating plasmon (presented in Fig. 2b) is defined as a ratio of the power flux of the propagating plasmon to the power flux of the electromagnetic wave incident onto the DGGGH period: $P = {{\int\limits_{0}^{L} {\int\limits_{ - \infty }^{ + \infty } {\left( {S_{x}^{ + } + S_{x}^{ - } } \right)dzdx} } } \mathord{\left/ {\vphantom {{\int\limits_{0}^{L} {\int\limits_{ - \infty }^{ + \infty } {\left( {S_{x}^{ + } + S_{x}^{ - } } \right)dzdx} } } {\left| {L^{2} \cdot S^{{{\text{inc}}}} } \right|}}} \right. \kern-\nulldelimiterspace} {\left| {L^{2} \cdot S^{{{\text{inc}}}} } \right|}},$ where $S_{x}^{ + }$ and $S_{x}^{ - }$ are the power fluxes of the plasma waves propagating along and against *x-*direction, respectively (see details in the Supplemental material), and $L$ is the length of the structure period. One can see that the transformation of the incident electromagnetic wave into the propagating plasmon in the regions of the map near the plasmon mode intercrossing is relatively high (Fig. 2b). Positive values of the transformation coefficient describe the transformation of the incident wave into the plasmon propagating along the *x*-direction (see red regions on the map), while negative values of this coefficient correspond to excitation of the plasmon travelling against the *x*-direction (blue regions on the map). Away the mode intercrossing regions, the incident electromagnetic wave excites rather weak plasmons in graphene. The transformation coefficient $P$ is equal almost to zero (regions on the map shown by white colour) away from the plasmon resonances. Near the mode intercrossing, the absolute value of the transformation coefficient can reach the unity and even exceed it (dark blue and dark red regions on the map). This is because of the gain provided by the active graphene.

Switching the plasmon propagation direction could be realized by two different methods of the dual grating gate voltage changing. As it is schematically shown in Fig. 1, the voltage with the value *U* is applied to the “left” subgrating, while the “right” subgrating is subjected to the voltage *V*. By interchanging the voltage values, i.e. applying voltage with the *V* value to the “left” subgrating and voltage with the value *U* to the “right” subgrating, we get plasmon propagating in the opposite direction with respect to the case of initially applied voltage values. The described above method of type *I* is illustrated in Fig. 2b. One can see that there are two mode intercrossings shown in the map. These mode intercrossings are symmetric with respect to the left-bottom to right-top regions diagonal. By swapping the voltage values in the subgratings (swapping the Fermi energy values in the upper graphene under the subgratings fingers) we obtain the region near the other mode intercrossing but the transformation coefficient changes from negative to positive value. There is another method of switching the plasmon propagation direction (type *II*) by changing the gate voltages values. The plasmon propagation direction could be switched when the Fermi energy value under one of the sub-gratings remains fixed while the other Fermi energy value is changed (by several meV) across the mode intercrossing (Fig. 2b). The propagating plasma wave can be easily transformed to a standing plasmon by equalizing the voltages *V* and *U* (point *III* in Fig. 2b). Let us discuss plasmon propagation switching methods of types *I* and *II* in detail.

We start from the method of type *II* of the plasmon propagation direction switching. This case allows for switching of the plasmon direction by a small change of the Fermi energy value. Figure 3 represents the transformation coefficient *P* (blue curve) in dependence on the Fermi energy value $$E_{{{\text{F1}}}}$$ under one of the subgratings for the fixed Fermi energy $$E_{{{\text{F2}}}} = 110$$ meV under the gates of the other subgrating. One can see that near the mode intercrossing the transformation of the incident electromagnetic wave into a propagating plasmon exceeds the unity due to the gain provided by the active graphene. We define so called positive (negative) unidirectional coefficients, which show the part of the plasmon power flux travelling along (against) the *x*-direction. Positive $$P_{( + )}^{{}}$$ and negative $$P_{( - )}^{{}}$$ unidirectional coefficients (which are always smaller than unity) are the ratios of the plasmon power fluxes propagating along and against the *x*-direction to the sum of the absolute values of these power fluxes:$${P_{( + )}} = {{\int\limits_0^L {\int\limits_{ - \infty }^{ + \infty } {S_x^ + dzdx} } } \mathord{\left/
 {\vphantom {{\int\limits_0^L {\int\limits_{ - \infty }^{ + \infty } {S_x^ + dzdx} } } {\int\limits_0^L {\int\limits_{ - \infty }^{ + \infty } {\left( {\left| {S_x^ + } \right| + \left| {S_x^ - } \right|} \right)dzdx} } }}} \right.
 \kern-\nulldelimiterspace} {\int\limits_0^L {\int\limits_{ - \infty }^{ + \infty } {\left( {\left| {S_x^ + } \right| + \left| {S_x^ - } \right|} \right)dzdx} } }};{P_{( - )}} = {{\int\limits_0^L {\int\limits_{ - \infty }^{ + \infty } {S_x^ - dzdx} } } \mathord{\left/
 {\vphantom {{\int\limits_0^L {\int\limits_{ - \infty }^{ + \infty } {S_x^ - dzdx} } } {\int\limits_0^L {\int\limits_{ - \infty }^{ + \infty } {\left( {\left| {S_x^ + } \right| + \left| {S_x^ - } \right|} \right)dzdx} } }}} \right.
 \kern-\nulldelimiterspace} {\int\limits_0^L {\int\limits_{ - \infty }^{ + \infty } {\left( {\left| {S_x^ + } \right| + \left| {S_x^ - } \right|} \right)dzdx} } }}$$. As one can see from Fig. 3, the positive (red curve) and negative (green curve) unidirectional coefficients can reach more than 80 percent, which means that more than 80 percent of the whole plasmon power flux propagates in one direction with the corresponding transformation coefficient (see Fig. 3, blue curve) substantially exceeding the unity (*P* exceeds 6 in value). For the method of type *I* of plasmon direction switching, the unidirectional coefficients values after swapping the voltage values corresponding to mode intercrossing remain as high as 80 percent while the transformation coefficient *P* exceeds 6. This could be seen from Fig. 3, taking into account the symmetry of the DGGGH with respect to swapping the voltage values under different subgratings of the dual grating gate.

## Discussion

We have shown the possibility to realize electrically tunable terahertz active plasmonic directional coupler based on the periodical DGGGH. Proposed directional coupler allows for switching the plasma wave propagation direction between two opposite directions along the graphene layers. Important features of this coupler is high efficiency of transformation of incident THz wave into propagating plasmon and high directionality of the plasmon due to using the dual-grating-gate and the active graphene to excite undumped and/or amplified propagating plasmons.

**Figure 4:**
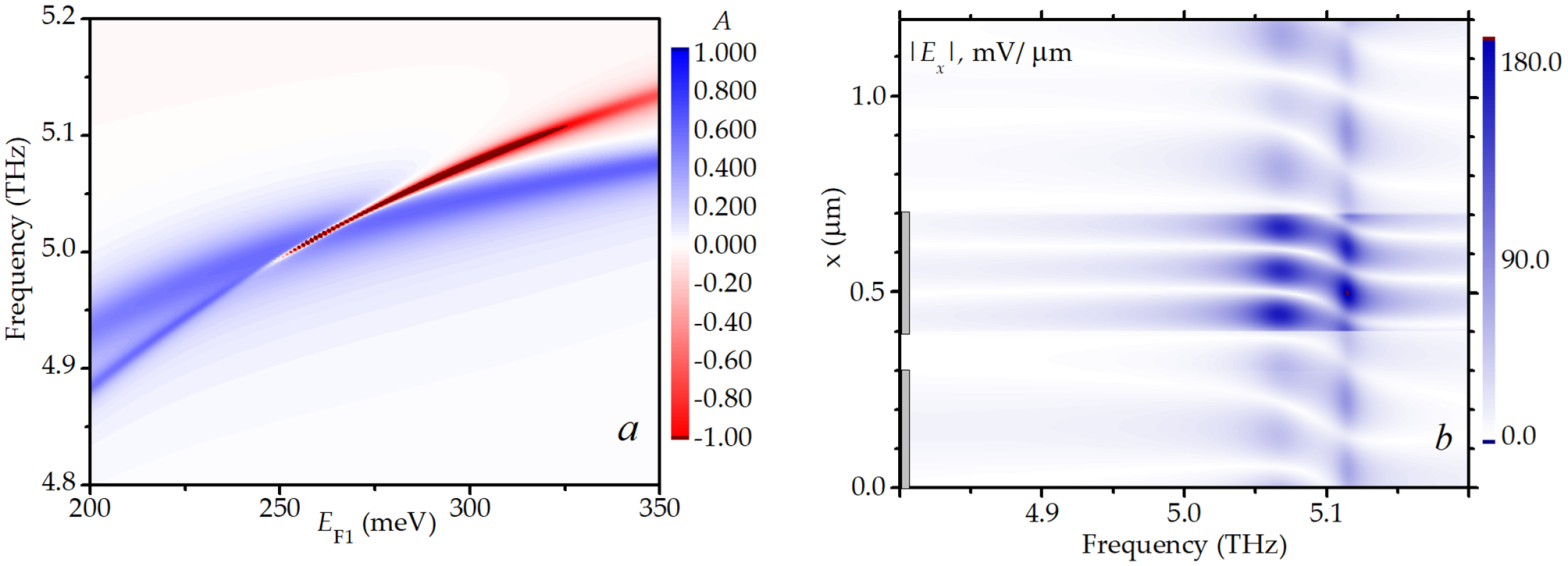
(**a**) Absorption coefficient vs the frequency and the Fermi energy in the upper graphene under one subgrating fingers with fixed value of Fermi energy $E_{{{\text{F2}}}} = 110$ meV under the other subgrating fingers. (**b**) The spatial distribution of the *x-*component of the electric field amplitude (absolute value) over the unit cell of the DGGGH in the passive graphene layer for the fixed Fermi energy values $E_{{{\text{F1}}}} = 330$ meV and $E_{{{\text{F2}}}} = 110$ meV a little shifted from the values corresponding to the plasmon mode intercrossing. The positions of the gate fingers are shown by the grey rectangles.

An important feature to be discussed is the structure of the plasmon modes near the intercrossing regimes where the switching of the plasmon propagation direction occur. In Fig. 4a we show the absorption coefficient versus the frequency and the Fermi energy in the upper graphene under one subgrating fingers with a fixed value of the Fermi energy $E_{{{\text{F2}}}} = 110$ meV under the other subgrating fingers (which corresponds to the plasmon mode intercrossing at frequency 5 THz). One can see a mode which absorption coefficient becomes negative at higher frequencies (due to the gain provided by the active graphene) and another mode with always positive absorption coefficient. These modes intercross at certain values of the frequency and Fermi energy. We present in panel Fig. 4b the spatial distribution of the *x-*component of the electric field amplitude over the unit cell of the DGGGH in the passive graphene layer for the fixed Fermi energy values a little shifted from the values corresponding to the plasmon mode intercrossing (those are $E_{{{\text{F1}}}} = 330$ meV and $E_{{{\text{F2}}}} = 110$ meV). For these Fermi energy values, the plasmon mode is mostly localized under the fingers of one subgrating. One can see two modes at frequencies above 5 THz. One of the modes has the odd number of the electric field antinodes under the gate fingers (“odd” plasmon mode) while the other mode has the even number of the electric field antinodes under the gate fingers (“even” plasmon mode). With increasing the frequency, the real part of the active graphene conductivity becomes negative and rises in its absolute value^31^. Therefore, at higher frequencies gain provided by the active graphene increases and compensates the losses of the even mode (the absorption coefficient of this mode becomes negative) for frequencies exceeding 5 THz. The higher-frequency plasmon mode in this case has a lower radiative damping, therefore it becomes an amplified mode due to the gain provided by the active graphene, while the other mode remains absorbing^39^.

Finally, the concept of the electrically controllable active plasmonic directional coupler of THz signal based on a periodical dual grating gate graphene structure is proposed. The advantage of effective energy transformation by using dual grating gate periodical graphene structure allows for excitation of unidirectionally propagating plasmon with great transformation efficiency and high directionality. One can control the plasmon propagation direction by applying different dc voltages to the subgratings of the grating gate. High transformation efficiency occurs due to amplification of plasmons in the structure by using pumped active graphene. Such electrically controllable directional coupler is of high importance for application in THz plasmonic integrated nanocircuits.

## Methods

Numerical calculations were performed using the electromagnetic approach based on the integral equation method. Basic steps of this approach are the following. (i) Maxwell equations, Ohm’s law and boundary conditions are written in the Fourier representation. (ii) The integral equations in respect of oscillating currents in the conducting areas of DGGGH are formed. (iii) The integral equations are numerically solved using the Galerkin procedure. The described method allows for calculation the field of plasma wave at any point of the structure. For more detail, see the Supplemental material.

## Supplementary Information

Supplementary Information.

## Data Availability

The data that support the findings of this study are available from the corresponding author upon request.
