# Supplementary material for: Electrically controllable active plasmonic directional coupler of terahertz signal based on a periodical dual grating gate graphene structure
Source: Sci Rep. 2021 Jun 1;11:11431. doi: 10.1038/s41598-021-90876-2 (PMC8169778; doi:10.1038/s41598-021-90876-2)
Supplement: Supplementary file 1 — Supplementary Information. [file 41598_2021_90876_MOESM1_ESM.pdf]

Electromagnetic approach

We studied the normal incidence of electromagnetic wave on the graphene structure (Fig. S1). The metal strips of the grating gate and graphene sheets were assumed being of zero thickness.

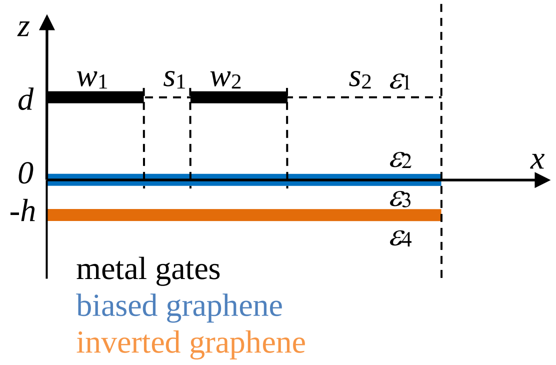

Fig.S1. Schematic view of one unit cell of periodic structure.

The electric field of the incident wave is polarized along the *x*-axis. All elements of the graphene structure are isotropic along the *y*-axis. The incident wave has only two components E_x^{inc} and H_y^{inc} which are the electric and magnetic fields, respectively. The incident wave excites scattered TM-polarized evanescent fields, which in Fourier representation can be written as $E_x\left(x,z,t\right)=\sum_{p=-\infty}^{\infty}e_{x,p}(z)\exp\left(i\left(q_p x-\omega t\right)\right)$, where E_x is the *x*-component of the electric field, $e_{x,p}(z)$ is the Fourier amplitude of the electric field, $q_p=\frac{2\pi p}{L}$ is *x*-component of the wave vector of *p*-th Fourier harmonics, and ω is the angular frequency.

The dependence of electric (and magnetic) fields on the z-coordinate could be represented as:

$$
\begin{aligned}
e_{x,p}^{(1)}(z)&=e_{x,p}^{(1)}\exp\left(iq_{z,p}^{(1)}z\right),\\
e_{x,p}^{(2)}(z)&=e_{x,p}^{(2,1)}\exp\left(iq_{z,p}^{(2)}z\right)+e_{x,p}^{(2,2)}\exp\left(-iq_{z,p}^{(2)}z\right),\\
e_{x,p}^{(3)}(z)&=e_{x,p}^{(3,1)}\exp\left(iq_{z,p}^{(3)}z\right)+e_{x,p}^{(3,2)}\exp\left(-iq_{z,p}^{(3)}z\right),\\
e_{x,p}^{(4)}(z)&=e_{x,p}^{(4)}\exp\left(iq_{z,p}^{(4)}z\right),
\end{aligned}
$$

where $e_{x,p}^{(n)}$ are Fourier amplitudes in different dielectric media, and $q_{z,p}^{(n)}$ are the *z*-components of wave vectors in different media. We used the standard boundary conditions at the boundaries of dielectric media

$$\begin{cases} e_{x,p}^{(1)}(d) + \delta_{p,0} E_x^{inc} = e_{x,p}^{(2)}(d), \\ h_{y,p}^{(1)}(d) + \delta_{p,0} H_y^{inc} - h_{y,p}^{(2)}(d) = -j_{x,p}(d), \end{cases}$$

$$\begin{cases} e_{x,p}^{(2)}(0) = e_{x,p}^{(3)}(0), \\ h_{y,p}^{(2)}(0) - h_{y,p}^{(3)}(0) = -j_{x,p}(0), \end{cases}$$

$$\begin{cases} e_{x,p}^{(3)}(-h) = e_{x,p}^{(4)}(-h), \\ h_{y,p}^{(3)}(-h) - h_{y,p}^{(4)}(-h) = -j_{x,p}(-h), \end{cases}$$

where $j_{x,p}(z)$ are the Fourier harmonics of the current densities in the planes $z = d$, $z = 0$, and $z = -h$.

The Fourier harmonics of the electric fields and current densities in spatially homogeneous graphene are connected by the Fourier-transformed Ohm's law as

$$j_{x,p}(-h) = \sigma_{pump}(\omega) \cdot e_{x,p}^{(3)}(-h) ,$$

where

$$\sigma_{pump}(\omega) = \frac{e^2 8 k_B T \tau}{4\pi\hbar^2 (1 - i\omega\tau)} \ln\left(1 + \exp\left(\frac{\varepsilon_F}{k_B T}\right)\right) + \frac{e^2}{4\hbar} \tanh\left(\frac{\hbar\omega - 2\varepsilon_F}{4 k_B T}\right) - \frac{e^2 \omega}{i\pi} \int_0^\infty \frac{G(\xi, \varepsilon_F) - G\left(\frac{\hbar\omega}{2}, \varepsilon_F\right)}{(\hbar\omega)^2 - 4\xi^2} d\xi,$$

is the conductivity of pumped graphene [1] and

$$G(\xi, \varsigma) = \frac{\sinh\left(\frac{\xi}{k_B T}\right)}{\cosh\left(\frac{\xi}{k_B T}\right) + \cosh\left(\frac{\varsigma}{k_B T}\right)},$$

ε_F is the quasi-Fermi energy $\varepsilon_F > 0$ for electrons and $\varepsilon_F < 0$ for holes), e is the elementary charge, τ is the intraband carrier relaxation time, k_B is the Boltzmann constant, and T is the temperature. The solution of Maxwell equations together with boundary condition in Fourier representation allows for writing the equations

$$e_{x,p}^{(2)}(d) = Z_p^{(1,1)} j_{x,p}(d) + Z_p^{(1,2)} j_{x,p}(0) + Z_{01} E_x^{inc} \qquad (1)$$

$$e_{x,p}^{(3)}(0) = Z_p^{(2,1)} j_{x,p}(d) + Z_p^{(22)} j_{x,p}(0) + Z_{02} E_x^{inc} \qquad (2)$$

$$e_{x,p}^{(4)}(-h) = Z_p^{(3,1)} j_{x,p}(d) + Z_p^{(3,2)} j_{x,p}(0) + Z_{03} E_x^{inc} \qquad (3)$$

where

$$Z_p^{(1,1)} = \frac{-q_{z,p}^{(1)} q_{z,p}^{(2)} \left(t_4 \left(q_{z,p}^{(2)} \varepsilon_3 t_6 t_8 + q_{z,p}^{(3)} \varepsilon_2 t_5 t_7 \right) - q_{z,p}^{(3)} t_1 \left(q_{z,p}^{(3)} \varepsilon_2 t_5 t_8 + q_{z,p}^{(2)} \varepsilon_3 t_6 t_7 \right) \right)}{\chi},$$

$$Z_p^{(1,2)} = Z_p^{(2,1)} = \frac{q_{z,p}^{(1)} \varepsilon_2 t_2 t_3 t_9}{\chi} \ ,$$

$$Z_p^{(2,2)} = -\frac{t_2 t_{10} t_9}{\chi},$$

$$Z_p^{(3,1)} = -t_4 t_{11} \frac{Z_p^{(1,2)}}{t_9},$$

$$Z_p^{(3,2)} = -t_4 t_{11} \frac{Z_p^{(2,2)}}{t_9},$$

$$\chi = \varepsilon_0 \omega \begin{pmatrix} t_6 \left(t_8 \left(q_{z,p}^{(1)} \left(q_{z,p}^{(3)} \right)^2 {\varepsilon_2}^2 t_1 + \left(q_{z,p}^{(2)} \right)^2 \varepsilon_1 \varepsilon_3 t_4 \right) - t_7 q_{z,p}^{(3)} \left(q_{z,p}^{(1)} {\varepsilon_2}^2 t_4 + \left(q_{z,p}^{(2)} \right)^2 \varepsilon_1 \varepsilon_3 t_1 \right) \right) \\ -t_5 q_{z,p}^{(2)} \varepsilon_2 \left(t_8 \left(q_{z,p}^{(1)} \varepsilon_3 t_4 + \left(q_{z,p}^{(3)} \right)^2 \varepsilon_1 t_1 \right) - t_7 \left(q_{z,p}^{(1)} q_{z,p}^{(3)} \varepsilon_3 t_1 + q_{z,p}^{(3)} \varepsilon_1 t_4 \right) \right) \end{pmatrix},$$

$$t_1 = q_{z,p}^{(4)} \sigma_{pump}(\omega) - \varepsilon_0 \varepsilon_4 \omega,$$

$$t_2 = q_{z,p}^{(2)} q_{z,p}^{(3)},$$

$$t_3 = 2 \exp\left(i d q_{z,p}^{(2)} \right),$$

$$t_4 = q_{z,p}^{(4)} \varepsilon_0 \varepsilon_3 \omega,$$

$$t_5 = 1 + \exp\left(2 i d q_{z,p}^{(2)} \right),$$

$$t_6 = -1 + \exp\left(2 i d q_{z,p}^{(2)} \right),$$

$$t_7 = 1 + \exp\left(2 i h q_{z,p}^{(3)} \right),$$

$$t_8 = -1 + \exp\left(2 i h q_{z,p}^{(3)} \right),$$

$$t_9 = -t_4 t_7 + q_{z,p}^{(3)} t_1 t_8,$$

$$t_{10} = q_{z,p}^{(2)} \varepsilon_1 t_6 - q_{z,p}^{(1)} \varepsilon_2 t_5,$$

$$t_{11} = 2 \exp\left(i h q_{z,p}^{(3)} \right),$$

$$Z_{01} = -\frac{2 e^{-i d q_{z,p}^{(1)}} \delta_{p,0} \varepsilon_0 \varepsilon_1 \omega}{q_{z,p}^{(1)}} Z_0^{(1,1)},$$

$$Z_{02} = -\frac{2 e^{-i d q_{z,p}^{(1)}} \delta_{p,0} \varepsilon_0 \varepsilon_1 \omega}{q_{z,p}^{(1)}} Z_0^{(1,2)},$$

$$Z_{03} = -\frac{2 e^{-i d q_{z,p}^{(1)}} \delta_{p,0} \varepsilon_0 \varepsilon_1 \omega}{q_{z,p}^{(1)}} Z_0^{(3,1)}.$$

Using equations (1) and (2) we create the system of six integral equations with respect to the unknown currents at the planes of metallic gates and at the plane of inhomogeneous graphene $z=0$:

$$j_x^{(w_\alpha)}(x,d)=\frac{\sigma_{w_\alpha}}{L}\sum_q Z_q^{[1,1]}\int_0^{w_1} j_x^{(w_1)}(x',d)\exp(-iqx')dx'\exp(iqx)$$
$$+\frac{\sigma_{w_\alpha}}{L}\sum_q Z_q^{[1,1]}\int_{w_1+s_1}^{w_1+s_1+w_2} j_x^{(w_2)}(x',d)\exp(-iqx')dx'\exp(iqx)$$
$$+\frac{\sigma_{w_\alpha}}{L}\sum_q Z_q^{[1,2]}\int_0^{w_1} j_x^{(w_1)}(x',0)\exp(-iqx')dx'\exp(iqx)$$
$$+\frac{\sigma_{w_\alpha}}{L}\sum_q Z_q^{[1,2]}\int_{w_1}^{w_1+s_1} j_x^{(s_1)}(x',0)\exp(-iqx')dx'\exp(iqx)$$
$$+\frac{\sigma_{w_\alpha}}{L}\sum_q Z_q^{[1,2]}\int_{w_1+s_1}^{w_1+s_1+w_2} j_x^{(w_2)}(x',0)\exp(-iqx')dx'\exp(iqx)$$
$$+\frac{\sigma_{w_\alpha}}{L}\sum_q Z_q^{[1,2]}\int_{w_1+s_1+w_2}^{L} j_x^{(s_2)}(x',0)\exp(-iqx')dx'\exp(iqx)+\sigma_{w_\alpha}Z^{[1]}E_x^{inc}\delta_{q,0},$$

$$j_x^{(w_\alpha)}(x,0)=\frac{\sigma_{g,(w_\alpha)}}{L}\sum_q Z_q^{[2,1]}\int_0^{w_1} j_x^{(w_1)}(x',d)\exp(-iqx')dx'\exp(iqx)$$
$$+\frac{\sigma_{g,(w_\alpha)}}{L}\sum_q Z_q^{[2,1]}\int_{w_1+s_1}^{w_1+s_1+w_2} j_x^{(w_2)}(x',d)\exp(-iqx')dx'\exp(iqx)$$
$$+\frac{\sigma_{g,(w_\alpha)}}{L}\sum_q Z_q^{[2,2]}\int_0^{w_1} j_x^{(w_1)}(x',0)\exp(-iqx')dx'\exp(iqx)$$
$$+\frac{\sigma_{g,(w_\alpha)}}{L}\sum_q Z_q^{[2,2]}\int_{w_1}^{w_1+s_1} j_x^{(s_1)}(x',0)\exp(-iqx')dx'\exp(iqx)$$
$$+\frac{\sigma_{g,(w_\alpha)}}{L}\sum_q Z_q^{[2,2]}\int_{w_1+s_1}^{w_1+s_1+w_2} j_x^{(w_2)}(x',0)\exp(-iqx')dx'\exp(iqx)$$
$$+\frac{\sigma_{g,(w_\alpha)}}{L}\sum_q Z_q^{[2,2]}\int_{w_1+s_1+w_2}^{L} j_x^{(s_2)}(x',0)\exp(-iqx')dx'\exp(iqx)+\sigma_{g,(w_\alpha)}Z^{[2]}E_x^{inc}\delta_{q,0},$$

$$
\begin{aligned}
j_x^{(s_\alpha)}(x,0) &= \frac{\sigma_{g,(s_\alpha)}}{L}\sum_q Z_q^{[2,1]}\int_0^{w_1} j_x^{(w_1)}(x',d)\exp(-iqx')dx'\exp(iqx) \\
&+\frac{\sigma_{g,(s_\alpha)}}{L}\sum_q Z_q^{[2,1]}\int_{w_1+s_1}^{w_1+s_1+w_2} j_x^{(w_2)}(x',d)\exp(-iqx')dx'\exp(iqx) \\
&+\frac{\sigma_{g,(s_\alpha)}}{L}\sum_q Z_q^{[2,2]}\int_0^{w_1} j_x^{(w_1)}(x',0)\exp(-iqx')dx'\exp(iqx) \\
&+\frac{\sigma_{g,(s_\alpha)}}{L}\sum_q Z_q^{[2,2]}\int_{w_1}^{w_1+s_1} j_x^{(s_1)}(x',0)\exp(-iqx')dx'\exp(iqx) \\
&+\frac{\sigma_{g,(s_\alpha)}}{L}\sum_q Z_q^{[2,2]}\int_{w_1+s_1}^{w_1+s_1+w_2} j_x^{(w_2)}(x',0)\exp(-iqx')dx'\exp(iqx) \\
&+\frac{\sigma_{g,(s_\alpha)}}{L}\sum_q Z_q^{[2,2]}\int_{w_1+s_1+w_2}^{L} j_x^{(s_2)}(x',0)\exp(-iqx')dx'\exp(iqx)+\sigma_{g,(s_\alpha)}Z^{[2]}E_x^{inc}\delta_{q,0},
\end{aligned}
$$

where indexes $\alpha = 1,2$ are used as designations for currents, which oscillate in the different conductive areas of the unit cell, for example, $j_x^{(s_1)}(x,0)$ is the current density in plane $z=0$ at a part of graphene under the narrow slit, that denoted by s_1. We introduce the designations σ_{w_α} for conductivity of metal gate fingers in THz frequencies and $\sigma_{g,(s_\alpha)}$ and $\sigma_{g,(w_\alpha)}$ are conductivities of homogeneous parts of inhomogeneous graphene layer (z = 0) in the unit cell. The conductivity of electrically biased parts of inhomogeneous graphene $\sigma_{g,(s_\alpha)}$ and $\sigma_{g,(w_\alpha)}$ was taken from [2]

$$
\sigma_g = \frac{e^2 8k_B T\tau}{4\pi\hbar^2(1-i\omega\tau)}\ln\left(2\cosh\left(\frac{E_{\mathrm{F}}}{2k_BT}\right)\right)+\frac{e^2}{4\hbar}G\left(\frac{\hbar\omega}{2},\varepsilon_F\right)-\frac{e^2\omega}{i\pi}\int_0^\infty \frac{G\left(\xi,E_{\mathrm{F}}\right)-G\left(\frac{\hbar\omega}{2},\varepsilon_F\right)}{\left(\hbar\omega\right)^2-4\xi^2}d\xi,
$$

E_F is the Fermi energy. We solved the system of integral equations by Galerkin method [3] with expansion of the current densities in the conductive parts of the unit cell of graphene structure into series of orthogonal Legendre polynomials

$$j_x^{(s_1)}(\rho_1,0)=\sum_{\beta=0}^{\infty}a_\beta^{(s_1)}P_\beta(\rho_1)$$

$$j_x^{(s_2)}(\rho_2,0)=\sum_{\beta=0}^{\infty}a_\beta^{(s_2)}P_\beta(\rho_2)$$

$$j_x^{(w_1)}(\rho_3,0)=\sum_{\beta=0}^{\infty}a_\beta^{(w_1,0)}P_\beta(\rho_3)$$

$$j_x^{(w_2)}(\rho_4,0)=\sum_{\beta=0}^{\infty}a_\beta^{(w_2,0)}P_\beta(\rho_4)$$

$$j_x^{(w_1)}(\rho_3,d)=\sum_{\beta=0}^{\infty}a_\beta^{(w_1,d)}P_\beta(\rho_3)$$

$$j_x^{(w_2)}(\rho_4,d)=\sum_{\beta=0}^{\infty}a_\beta^{(w_2,d)}P_\beta(\rho_4)$$

where

$$\rho_1=2\frac{x-w_1}{s_1}-1,$$

$$\rho_2=2\frac{x-L}{s_2}+1,$$

$$\rho_3=\frac{2x}{w_1}-1,$$

$$\rho_4=2\frac{x-s_1-w_1}{w_2}-1,$$

$a_\beta^{(w_\alpha)}$ and $a_\beta^{(w_\alpha,0)}$ are the expansion coefficients, $P_\beta(\rho)$ are Legendre polynomials. Substitution of expansions into the system of integral equations transforms it into an infinite system of linear algebraic equations for coefficients a_β. Based on the convergence of this numerical procedure, we truncated the system of the algebraic equations and solved it numerically. Finally, upon finding the current densities by solving Eq. (1-3) and using Maxwell equations in each media, we calculated the induced electric and magnetic fields at an arbitrary point of the structure. The power fluxes of the incident, reflected, transmitted and plasma waves were calculated via the following formula $\mathbf{S}=\frac{1}{2}[\mathbf{EH}^*]$.

The power fluxes in the considered graphene structure can be divided into electromagnetic wave power fluxes and plasma wave power fluxes. The power of an electromagnetic wave can be calculated as the projection of the vector $\mathbf{S}$ onto the *z*-axis: $S^{inc}=-\frac{1}{2}\sqrt{\frac{\varepsilon_1\varepsilon_0}{\mu_0}}\left|E_x^{inc}\right|^2$ is the incident power flux, $S^{inc}=\frac{1}{2}\sqrt{\frac{\varepsilon_1\varepsilon_0}{\mu_0}}\left|e_{x,0}^{(1)}\right|^2$ is the reflected power flux, and

$S^T = -\frac{1}{2}\sqrt{\frac{\varepsilon_4\varepsilon_0}{\mu_0}}\left|e_{x,0}^{(4)}\right|^2$ is the transmitted power flux. The plasma wave power flux can be calculated as the projection of the vector $\mathbf{S}$ onto the *x*-axis – S_x, this flow consists of power flows along the *x*-axis S_x^+ and against the *x*-axis S_x^-.

References

1. A. A. Dubinov, *et al.* Terahertz surface plasmons in optically pumped graphene structures. *J. Phys.: Condens. Matter.* 23, 145302 (2011).

2. M. S. Jang, *et al.* Tunable large resonant absorption in a midinfrared graphene salisbury screen. *Phys. Rev. B* 90, 165409 (2014).

3. Computational Galerkin Methods, edited by J. Fletcher (Springer, New York, 1984).
